# Community detection in networks by dynamical optimal transport formulation

**DOI:** 10.1038/s41598-022-20986-y

**Published:** 2022-10-07

**Authors:** Daniela Leite, Diego Baptista, Abdullahi A. Ibrahim, Enrico Facca, Caterina De Bacco

**Affiliations:** 1 Max Planck Institute for Intelligent Systems, Cyber Valley, 72076 Tübingen, Germany; 2 Univ. Lille, Inria, CNRS, UMR 8524 - Laboratoire Paul Painlevé, 59000 Lille, France

**Keywords:** Applied mathematics, Complex networks, Computational science

## Abstract

Detecting communities in networks is important in various domains of applications. While a variety of methods exist to perform this task, recent efforts propose Optimal Transport (OT) principles combined with the geometric notion of Ollivier–Ricci curvature to classify nodes into groups by rigorously comparing the information encoded into nodes’ neighborhoods. We present an OT-based approach that exploits recent advances in OT theory to allow tuning between different transportation regimes. This allows for better control of the information shared between nodes’ neighborhoods. As a result, our model can flexibly capture different types of network structures and thus increase performance accuracy in recovering communities, compared to standard OT-based formulations. We test the performance of our algorithm on both synthetic and real networks, achieving a comparable or better performance than other OT-based methods in the former case, while finding communities that better represent node metadata in real data. This pushes further our understanding of geometric approaches in their ability to capture patterns in complex networks.

## Introduction

Complex networks are ubiquitous, hence modeling interactions between pairs of individuals is a relevant problem in many disciplines^1,2^. Among the variety of analyses that can be performed on them, community detection^3–6^ is a popular application that involves finding groups (or communities) of nodes that share similar properties. The detected communities may reveal important structural properties of the underlying system. Community detection has been used in diverse areas including, discovering potential friends on social networks^7^, evaluating social networks^8^, personalized recommendation of item to user^9^, detecting potential terrorist activities on social platforms^10^, fraud detection in finance^11^, study epidemic spreading process^12^ and so on.

Several algorithms have been proposed to tackle this problem which utilize different approaches, such as statistical inference^13,14^, graph modularity^15^, statistical physics^16^, information theory^17^ and multifractal topological analysis^18^. Here, instead, we adopt a recent approach connecting community detection with geometry, where communities are detected using geometric methods like the Ollivier–Ricci curvature (ORC) and we exploit a dynamical approach of optimal transport theory to calculate this efficiently and flexibly across various transportation regimes.

In Riemannian geometry, the sign of the curvature quantifies how geodesic paths converge or diverge. In networks, the ORC plays a similar role: edges with negative curvature are traffic bottlenecks, whereas positively curved ones allow mass to flow more easily along the network. Defining communities as structures that allow robust transport of information, we could cluster edges based on their curvature: those with positive curvature can be clustered together, while those with negative curvature may be seen as “bridges” connecting different communities. The idea of using Ricci curvature to find communities on networks was first proposed by Jost and Liu ^19^ and then further explored in subsequent works^20–23^. Our work follows a similar approach as in^22,23^ to calculate the OR curvature, but generalizes it for the cases of branched^24,25^ and congested^26^ optimal transport problems, building from recent results^27,28^. Specifically, our algorithm allows to efficiently tune the sensitivity to detecting communities in a network, through a parameter that controls the flow of information shared between nodes. We perform a comprehensive comparison between the proposed algorithm and existing ones on synthetic and real data. Our algorithm, named ORC-Nextrout, detects communities in synthetic networks with similar or higher accuracy compared to other OT-based methods in the regime where inference is not trivial, i.e. the inference problem is neither too easy nor too difficult to solve, and thus communities are only partially retrieved. This is also observed in a variety of real networks, where the ability to tune between different transportation regimes allows finding at least one result that outperforms other methods, including approaches based on statistical inference and modularity.

### Related work

The idea of exploring the geometrical properties of a graph, and in particular curvature, has been explored in different branches of network science, ranging from biological^29^ to communication^30^ networks. Intuitively, the Ricci curvature can be seen as the amount of volume through which a geodesic ball in a curved Riemannian manifold deviates to the standard ball in Euclidean spaces^31^. When defined in graphs, it indicates whether edges (those with positive values for the curvature) connect nodes inside a cluster, or if they rather bond different clusters together (those with negative values for the curvature).

Previous works^32–35^ extended the idea of the OR curvature. In^32^, the authors introduced the concept of “resistance curvature” for both nodes and edges. Taking inspiration from electrical circuits, this approach assigns a resistance being applied by the whole network from a current that flows between any two edges and correlates this to known concepts of OR discrete curvature. The resistance curvature provides a natural way to define the Ricci flow. In^33^ the authors proposed a *dynamical* version of the OR curvature, where a continuous-time diffusion process is defined for every node, at different time scales. In this context, the dynamical perspective is used to frame probability masses at nodes in terms of diffusion processes, e.g. those deployed in random walks. In our work instead, the dynamics enters to solve efficiently the underlying optimization problem required to compute the OR curvature. Regardless of the choice of the distribution that characterizes mass on nodes, this quantity is then used to define the curvature of the edges of the graph. Previous works have typically defined the OR curvature in terms of the 1-Wasserstein distance. In contrast, we take a more general approach and explore the usage of the $\beta$-Wasserstein, where $\beta \in (0,2]$, to account for a variety of OT problems, ranging from branched to congested transportation.

Other discrete graph curvature approaches include the Ollivier–Ricci (OR) curvature based on the Optimal Transport theory introduced by Ollivier,^36,37^ and Forman–Ricci curvature introduced by Forman^38^. While the graph Laplacian-based Forman curvature is computationally fast and less geometrical, we focus on the OT-based approach due to its more geometric nature. Some applications of the Ollivier–Ricci curvature include network alignment^39^ and community detection^22,23,40^.

On the other hand, community detection in networks is a fundamental area of network science, with a wide range of approaches proposed for this task^3,4,41^. These include methods based on statistical mechanical models^16,17,42^, probabilistic generative models^13,43–45^, nonnegative matrix factorization^46^, spectral methods^47,48^, multifractal topological analysis^18^ and modularity optimization^15,49,50^. In contrast, our work is inspired by recent OT-based methods^22,23^ for community detection. These methods consider the OR curvature to sequentially identify and prune negatively curved edges from a network to identify communities. While our approach also considers OR curvature to prune edges, it controls the flow of information exchanged between nodes by means of a traffic-penalization parameter, making the edge pruning completely dynamic. This is detailed in “β-Wasserstein community detection algorithm” section.

## β-Wasserstein community detection algorithm

In this section, we describe how our approach solves the community detection problem. As previously stated, we rely on optimal transport principles to find the communities. To solve the optimal transport problem applied in our analysis we use the discrete *Dynamic Monge-Kantorovich* model (*DMK*), as proposed by Facca et al.^51,52^ to solve transportation problems on networks.

We denote a weighted undirected graph as $G = (V,E, W)$, where *V*, *E*, *W* are the set of nodes, edges, and weights, respectively. We use the information of the neighborhood of a node *i*, $\mathscr{N}(i) = \{j \in V | (i,j) \in E\},$ to decide whether a node belongs to a given community. We do this by comparing a distribution defined on $\mathscr{N}(i)$ with those defined on other nodes close to *i*. There are several choices that can be made for this. For instance, one could frame this in the context of diffusion processes on networks and relate the distribution to random walkers traveling along the network with a certain jump probability^33^. Here we follow previous work^39^ and assign it as $m_i^{\alpha }$, where $m^{\alpha }_i(k):= \alpha$ if $k=i$ and $m^{\alpha }_i(k):= (1-\alpha )/|\mathscr{N}(i)|$ if $k \in \mathscr{N}(i)$. Intuitively, the distribution *m* assigns a unit of mass to *i* and its connections: $\alpha$ controls how much weight node *i* should have, and once this is assigned, its neighbors receive the remaining mass in an even way. We use $\alpha =0$ in all the experiments reported in this manuscript, i.e. the mass is equally distributed on the neighbors. This corresponds to a one-step transition probability for a random walker in the context of diffusion processes.

The next step is to compare the distribution $m_i^{\alpha }$ of node *i* to that of its neighbors. Consider an edge $(i,j)\in E$ and $m_j$, the distribution defined on node *j*, neighbor of *i*. We assume that if *i* and *j* belong to the same community, then both nodes may have several neighbors in common and, therefore, $m_i$ and $m_j$ should be similar. Note that this is valid for both assortative and disassortative community structures. In the former case, nodes are more likely to interact within the same community, while in the latter case we have the opposite, nodes are more likely to interact across communities^2,4^. When there is a consistent community pattern for all groups (e.g. all communities are assortative), this idea of comparing the distributions $m_i^{\alpha }$ may be appropriate to detect communities. On the contrary, it may be difficult to perform this task in networks with mixed connectivity patterns, where some communities are assortative and others are disassortative. This makes it difficult to detect communities as edges within an assortative community are shortened, likewise edges between a node in a disassortative and a node in an assortative one. This may confuse the algorithm, as both types of edges are shortened. A careful treatment of these cases is an interesting direction for future work.

**Figure 1:**
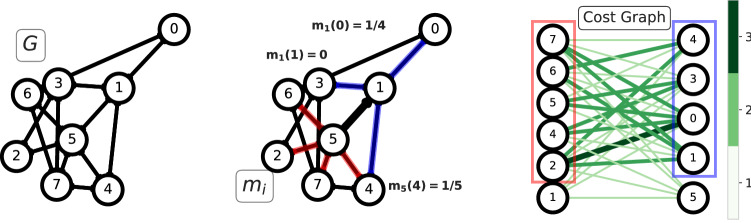
Left: an example graph *G* where edges have unitary weights. Center: the edge (1, 5) (bold black line) is selected to define the OT problem between $m_{1},m_{5}$; neighborhoods of nodes 1 and 5 are highlighted with blue and red edges and are used to build the corresponding distributions $m_{1},m_{5}$. Right: The complete bipartite graph $B_{15}$ where the OT problem is defined. The color intensity of the edges represents the distance between the associated nodes on the graph *G*, as shown by the color bar. $m_1$ and $m_5$ are both defined for $\alpha =0,$ i.e. no mass is left in 1 and 5.

To estimate the similarity between $m_i$ and $m_j$ we use OT principles. Specifically, we compute the cost of transforming one distribution into the other. This is related to the cost of moving the mass from one neighborhood to the other, and it is assumed to be the weighted shortest-path distance between nodes belonging to $\mathscr{N}(i)$ and $\mathscr{N}(j)$. A schematic representation of the algorithm can be seen in Fig. 1. The OT problem is solved in an auxiliary graph, the complete bipartite network $B_{ij} = (V_{ij}, E_{ij}, \omega _{ij})$ where $V_{ij} := (V_{i},V_{j}) :=(\mathscr{N}(i) \cup \{i\}, \mathscr{N}(j) \cup \{j\})$, $E_{ij}$ is made of all the possible edges between $V_{i}$ and $V_{j}$. The weights of the edges are given by the weighted shortest path distance *d* between two nodes measured on the input network *G*.

**Figure 2:**
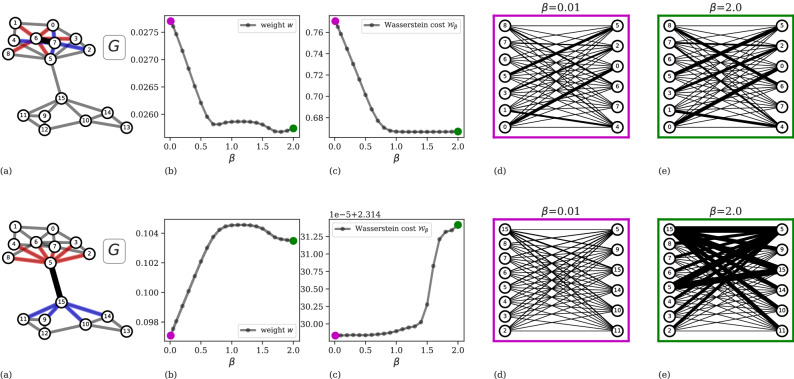
Visualization of how $\beta$ impacts intra-community and inter-community edge weights. (**a**) Examples of intra-community (top panel) and inter-community (bottom panel) structures between nodes 6 and 7, and nodes 5 and 15, respectively. (**b**) The weight of edge (6, 7) decreases when $0<\beta <0.6$, while for $0.5<\beta <2.0$ it reaches a minimum, and then slightly increases again. Similar but opposite pattern is observed for the edge (5, 15). (**c**) The $\beta$-Wasserstein cost: for intra-community edges, $\beta >1$ consolidates traffic in the network as the Wasserstein cost stabilizes, making it minimum for the extreme value $\beta =2$, whereas it is maximized in the case of the inter-community edge. (**d**,** e**) Example cost graphs $B_{67}$ (top) and $B_{515}$ (bottom) with fluxes solution of the OT problem (edge thickness is proportional to the amount of flux) in the regimes of small (**d**) and high (**e**) values of $\beta$.

The similarity between $m_i$ and $m_j$ is the Wasserstein cost $\mathscr{W}(m_i,m_j,\omega _{ij})$ of the solution of the transportation problem. In its standard version, this number is the inner product between the solution *Q*, a vector of flows defined on edges, and the cost $\omega _{ij}$. In our case, since the DMK model allows to control the flow of information through a hyperparameter $\beta \in (0,2]$, we define the $\beta$*-Wasserstein cost*, $\mathscr{W}_\beta (m_i,m_j,\omega _{ij})$, as the inner product of the solution $Q =Q(\beta )$ of the DMK model and the cost $\omega _{ij}$. For $\beta =1$ we compute the $1-$Wasserstein distance between $m_i$ and $m_j$, while for $\beta \ne 1$ the influence of $\beta$ in the solution of the transportation problem can be seen in Fig. 2. When $\beta <1,$ more edges of *B* tend to be used to transport the mass, thus we observe congested transportation^26^. When $\beta >1$ fewer edges are used, hence we observe branched transportation, and the $\beta$*-Wasserstein cost* coincides with a branched transport distance^25,53^. The idea of tuning $\beta$ to interpolate between various transportation regimes has been used in several works and engineering applications^27,54–59^.

Calculating the Wasserstein cost is necessary to determine our main quantity of interest, the discrete Ollivier–Ricci curvature, defined as

1 $$\begin{aligned} \kappa _\beta (i,j) := 1 - \dfrac{\mathscr{W}_\beta (m_i,m_j,\omega _{ij})}{d_{ij }} \quad , \end{aligned}$$

where $d_{ij}$ is the weighted shortest path distance between *i* and *j* as measured in *G*. Intuitively, if *i* and *j* are in the same communities, several $k \in V_{i}$ and $\ell \in V_{j}$ will be also directly connected. Thus, the Wasserstein distance between $m_{i}$ and $m_{j}$ will be shorter than $d_{ij}$, yielding a positive $\kappa _\beta (i,j)$. Instead, when *i* and *j* are in different communities, their respective neighbors will be unlikely connected, hence $d_{ij}<\mathscr{W}_\beta (m_i,m_j,\omega _{ij})$, yielding a negative $\kappa _\beta (i,j)$.

The Ricci flow algorithm on a network is defined by iteratively updating the weights of the graph *G*^22,23^. These are updated by combining the curvature and shortest path distance information^36^. We redefine these updates using our proposal for the Ollivier–Ricci curvature:

2 $$\begin{aligned} w_{ij}^{(t+1)} := d_{ij}^{(t)} - \kappa _\beta ^{(t)}(i,j)\cdot d_{ij}^{(t)}, \end{aligned}$$

where $w_{ij}^{(t+1)}$ is the weight of edge (*i*, *j*) at time *t*, $w_{ij}^{(0)} = d_{ij}^{(0)},$ and $d_{ij}^{(t)}$ is the shortest path distance between nodes *i* and *j* at iteration *t*. At every time step *t*, the weights are normalized by their total sum.

The algorithm ORC-Nextrout dynamically changes the weights of the graph *G* to isolate communities: intra-community edges will be shortened, while inter-community ones will be enlarged. These changes are reached after a different number of iterations of the whole routine depending on the input data. To find the communities, we apply a *network surgery* criterion on the edges based on the stabilization of the modularity of the network, as proposed by Ni et al.^22^. Notice that our algorithm does not need prior information about the number of communities: edges will be enlarged or shortened depending on the optimal transport principles, agnostic to community labeling. The computational complexity of the algorithm is dominated by that of solving the DMK model, which takes $O(|E|^{2.36})$ (estimated numerically) and by computing weighted shortest path distances $d_{ij}$, which costs $O(|V|^2\log |V|+|V||E|)$^60^. A pseudo-code of the implementation is shown in Algorithm 1.
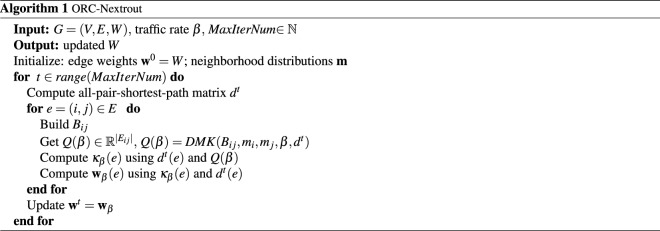


## Results on community detection problems

### Synthetic networks

To investigate the accuracy of our model in detecting communities, we consider synthetic networks generated using the *Lancichinetti–Fortunato–Radicchi* (LFR) benchmark^61^ and the *Stochastic Block Model* (SBM)^62^. Both models provide community labels used as *ground-truth* information during the classification tasks.

*Lancichinetti–Fortunato–Radicchi benchmark:* this benchmark generates undirected unweighted networks *G* with disjoint communities. It samples node degrees and community sizes from power law distributions, see Fig. 4 for an example. One of its advantages is that it generates networks with heterogeneous distributions of degrees and community sizes. The main parameters in input are the number of nodes *N*,  two exponents $\tau _1$ and $\tau _2$ for the power law distributions of the node degree and community size respectively, the expected degree *d* of the nodes, the maximum number of communities on the network $K_{max}$ and a fraction $\mu$ of inter-community edges incident to each node. To test the performance of our algorithm, we use the set of LFR networks used and provided by the authors of^22^. We set $\tau _1=2,$ $\tau _2 = 1,$ $d=20,$ $K_{max}=50$ and $\mu \in [0.05,0.75]$.

*Stochastic Block Model:* this model probabilistically generates networks with non-overlapping communities. One specifies the number of nodes *N* and the number of communities *K*, together with the expected degree *d* of a node and a ratio $r\in [0,1]$. Networks are generated by connecting nodes with a probability $r*p_{intra}$ if they belong to different communities; $p_{intra}$ if they are part of the same community, where $p_{intra} = d\times K/N$. Notice that the smaller the ratio *r* is, the fewer inter-community connections would exist, which leads to networks with a more distinct community structure.

We set $N=500$, $K=3$, $d=15$ and $r\in [0.01, 0.5]$ and generate 10 random networks per value of *r*.

**Results.** To evaluate the performance of our method in recovering the communities, we use the *Adjusted Rand Index* (ARI)^63^. ARI compares the community partition obtained with the *ground truth* clustering. It takes values ranging from 0 to 1, where ARI $=0$ is equivalent to random community assignment, and ARI $=1$ denotes perfect matching with the ground truth communities, hence the higher this value, the better the recovery of communities. A more detailed presentation of this metric is given in the Supplementary Information.

We test our algorithm for different types of information spreading in our OT-based model, as controlled by the parameter $\beta$, using the software developed in^64^ (available at https://gitlab.com/enrico_facca/dmk_solver). We used $\beta = 1$, i.e., standard Wasserstein distance; $\beta \in \{0.1,0.5\}$ for congested transportation, enforcing broad spreading across neighbors; and $\beta \in \{1.5,2 \}$ to favor branching schemes, where fewer edges are used to decide which community a node should belong to. For OT-based algorithms where we update the weights in Eq. (2) for 15 times (${\textit{MaxIterNum}}=15$ in Algorithm 1). Since in some cases the ARI score does not consistently increase with the number of iterations, we show results only for the iteration that maximizes the score.

**Figure 3:**
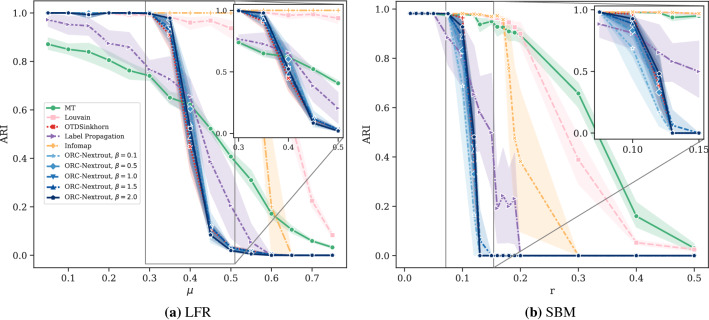
Results on LFR and SBM synthetic data. Performance in detecting ground-truth communities is measured by the ARI score. Markers and shadows are the averages and standard deviations over 10 network realisations with the same value of the parameter used in generation. Markers’ shape denote different algorithms. (**a**) LFR graph with $N=500$ nodes and different values of *K* ranging from (17, 22). (**b**) SBM with $N=500$ nodes, $K=3$ communities and average degree $d=15$. The parameter *r* is the ratio of inter-community with intra-community edges. The inset on each plot zooms in the central parts of the plots.

**Figure 4:**
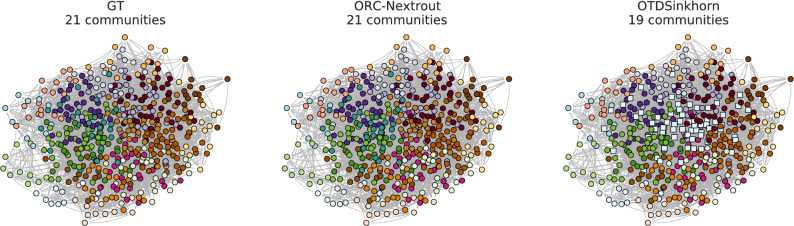
Example of community structure on a synthetic LFR network. The rightmost panel shows the ground-truth community structures to be predicted in an LFR network generated using $\mu = 0.35$. This network is one sample of the synthetic data used in Fig. 3. Square-shaped markers denote nodes that are assigned to communities different from those in ground-truth. In the middle and last panels, ORC-Nextrout with $\beta =2$ perfectly retrieves the 21 communities, while OTDSinkhorn predicts only 19 communities with an ARI score of 0.73, wrongly assigning ground-truth dark green and light brown (square-shaped) nodes to the light blue community.

The results in Fig. 3 show the performance on both LFR and SBM benchmarks with OT-based methods, our method for various $\beta$ and one based on the Sinkhorn algorithms (OTDSinkhorn)^65,66^. Our main goal is to assess the impact of tuning between different transportation regimes (as done by $\beta$) in terms of community detection via OT principles. Nevertheless, to better contextualize the performance of OT-based algorithms in the wide spectrum of community detection methods, we also include comparisons with algorithms that are not OT-based. Namely, we consider a probabilistic model with latent variables (MT)^13^, two modularity-based algorithms, Label Propagation^14^ and Louvain^50^, and with a flow-based algorithm, Infomap^17^. Our algorithm outperforms OTDSinkhorn for various values of $\beta$ in an intermediate regime where OT-based inference is not trivial, i.e. detecting communities is neither too easy nor too difficult. This occurs in both the LFR and SBM benchmark, as shown in Fig. 3. For lower and higher values of the parameters, performance is similar and close to the two extremes of $\hbox {ARI} = 0$ and 1. OT-based methods have a similar sharp decay in performance from the regime where inference is easy to the more difficult one, as also observed in^22^. The other community detection methods have smoother decay, but with lower performance in the regime where OT-based approaches strive, except for Label Propagation and MT, which are more robust in this sense. In the intermediate regime where inference is not trivial (i.e. along the sharp decay of OT-based methods), we observe that different values of $\beta$ give higher performance than OTDSinkhorn in most cases. For SBM the highest performance is consistently achieved for high $\beta =2$, while for LFR the best $\beta$ varies with $\mu$. A qualitative example where ORC-Nextrout is performing better than OTDSinkhorn, in an instance of LFR of this intermediate regime, is shown in Fig. 4. Note that in this case, ORC-Nextrout perfectly recovers the 21 communities described by the ground-truth network, whereas OTDSinkhorn merges three of the central communities into one group, therefore recovering only 19 groups.

These results suggest that practitioners may choose the $\beta$ that gives the best performance in detecting communities, e.g. the one that maximizes ARI or other relevant metrics depending on the application at hand. We show examples of this on real data below.

### Analysis of real networks

Next, we evaluate our model on various real datasets^67^ containing node metadata that can be used to assess community recovery. While failing to recover communities that align well with node metadata should not be automatically interpreted as a model’s failure^68^ (e.g. the inferred communities and the chosen node metadata may capture different aspect of the data), having a reference community structure to compare against allows one to inspect quantitatively difference between models. These real networks differ on structural features like number of nodes, average degree, number of communities, and other standard network properties as detailed in Table 1. Specifically, we consider (i) a network of co-appearances of characters in the novel *Les Misérables*^69^ (Les Miserables). Edges are built between characters that encounter each other. (ii) A network of 62 bottlenose dolphins in a community living off Doubtful Sound, in New Zealand^70^ (Dolphins). The nodes represent dolphins, and the edges indicate frequent associations between them. This network is clustered into four groups, conjectured as clustered from one population and three sub-populations based on the interactions between dolphins of different sex and ages^71^. The dolphins were observed between 1994 and 2001. (iii) A network of Division I matches of American Football during a regular season in the fall of 2000^49^ (American football). Nodes represent teams, and edges are games between teams. Teams can be clustered according to their football college conference memberships. (iv) A network of books on US politics published around the 2004 presidential election and sold by an online bookseller^72^ (Political books). Nodes represent the books, and the edges between books are frequent co-purchasing of books by the same buyers. Books are clustered based on their political spectrum as neural, liberal, or conservative.

**Table 1:** Real networks description. We report statistics for the real networks used in our experiments. *N* and *E* denote the number of nodes and edges, respectively. *K* is the number of communities in the ground truth data. AvgDeg, AvgBtw and AvgClust are the average degree, betweenness centrality and average clustering coefficient, respectively.

Dataset	*N*	*E*	*K*	AvgDeg	AvgBtw	AvgClust
Les Miserables	77	254	11	6.6	0.0219	0.5731
Dolphins	62	159	4	5.1	0.0393	0.2590
American football	115	613	12	10.7	0.0133	0.4032
Political books	105	441	3	8.4	0.0202	0.4875

**Figure 5:**
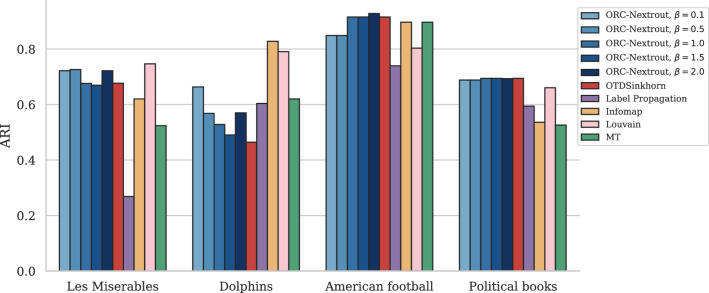
Results on real data. Performance in terms of recovering communities using metadata information is calculated in terms of the ARI score. ORC-Nextrout shows competing results against all methods with different optimal $\beta$ across datasets.

**Figure 6:**
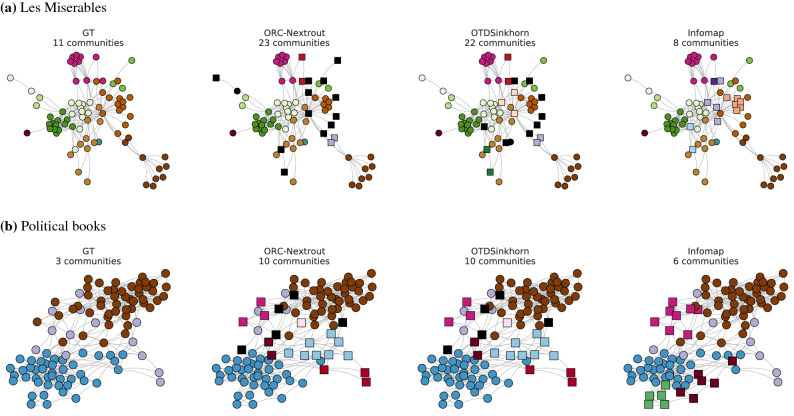
Communities in real networks. We show the communities inferred for Les Miserables (**a**) and Political books (**b**) by ORC-Nextrout ($\beta =0.5,0.1$ for top and bottom rows respectively), OTDSinkhorn and Infomap and compare against those extracted using node attributes (GT). The visualization layout is given by the *Fruchterman-Reingold force-directed* algorithm^73^, therefore, groups of well-connected nodes are located close to each other. Dark nodes represent individual nodes who are assigned to isolated communities by OT-based methods. Square-shaped markers denote nodes assigned to communities different from those obtained from node metadata.

OT-based algorithms outperform other community detection algorithms in detecting communities aligned with node metadata for two of the four studied datasets, as shown in Fig. 5. In particular, ORC-Nextrout has the highest accuracy performance considering the best performing $\beta$ in these cases. The impact of tuning this parameter is noticeable from these plots, as the best-performing value varies across datasets. In Les Miserables and Dolphins networks, $\beta <1$ has better performance, while in American Football the best performing value is for $\beta >1$. Performance is similar across OT-based methods in the Political books network. In Fig. 6 we show the communities detected by the best-performing ORC-Nextrout version together with OTDSinkhorn and Infomap in Les Miserables and Political books (see Supplementary Material for the remaining datasets). Focusing on Les Miserables, we see how ORC-Nextrout successfully detects three characters in the green communities, in particular a highly connected node in the center of the figure (in dark green). Notice that these are placed in the same community (pink or black) by OTDSinkhorn. Thus ORC-Nextrout achieves a higher ARI than OTDSinkhorn. Both OT-based approaches retrieve well communities exhibiting clustering patterns with many connections within the community. Instead, they both divide the communities with a hub and spokes structure due to the lack of common connections within the group.

The communities detected in both datasets highlight the tendency of OT-based methods to extract a larger number of communities than those observed from node metadata. Among these extra communities, some are made of a few nodes (e.g. the light-blue and violet), while others are made of one isolated node each (highlighted in black). This is related to the fact that OT-based methods perform particularly well for networks with internally densely connected community structures, but may be weaker for community structures that are sparsely connected^23^. One could potentially assign these nodes to larger communities, for instance, by preferential attachment as done in^23^, thus in practice reducing the number of communities. Devising a principled method or criterion to do this automatically is an interesting topic for future work. This tendency is further corroborated by the fact that OT-based algorithms recover robustly the two communities that are mostly assortative (blue and brown in the figure) in the Political books network, while they struggle to recover the disassortative community depicted in the center (violet). This community has several connections with nodes in the other two communities and has been separated into smaller groups by OT-based approaches, as described above. This also highlights the need for methods that are robust against situations where mixed connectivity patterns arise, i.e. a combination of assortative and disassortative communities coexisting in a network.

### Two tests on semi-synthetic networks

To further investigate the different performance gaps between the various approaches, we expand the comparison between the OT-based methods and Infomap on two semi-synthetic scenarios generated from Les-Miserables (Fig. 6a) and Dolphins datasets, where the largest ARI differences were observed. Specifically, we add random noise to the existing set of connections to understand if the performance gap can also be observed in more challenging scenarios. We add noise to the real data in two different ways (more details can be found in the Supplementary Material):

1. *Flipping entries:* from a given network, we generate a new one by flipping *R* entries of its adjacency matrix *A* uniformly at random. This means that if $A_{ij}=1$, this is changed to $A_{ij}=0$, and vice versa. The flipping of an entry $A_{ij}$ occurs with probability $p = 0.1$.
2. *Removing intra-community edges:* from a given network, we build a new one with the same inter-community structure but modified intra-community one by removing *R* within-community edges uniformly at random, based on the ground truth communities. To avoid generating disconnected networks, we only sample edges that are not connected to any leaves.

Both of these procedures make inference harder, but they act differently. The first process is meant to add random noise independently of the community structure (flips are made uniformly at random), while the second aims at targeting the community structure by weakening the assortative structure. We choose *R* to be $r\times |V|^2$ for the first test and $r\times |E|$ for the second, where we vary $r\in [0,1]$ to study the impact of these parameters on inferring the communities as measured by the ARI score. We generate 20 samples for each of these two mechanisms built using the Les-Miserables dataset ($|V|=77$, $|E|=254$) shown in Fig. 6a.

**Figure 7:**
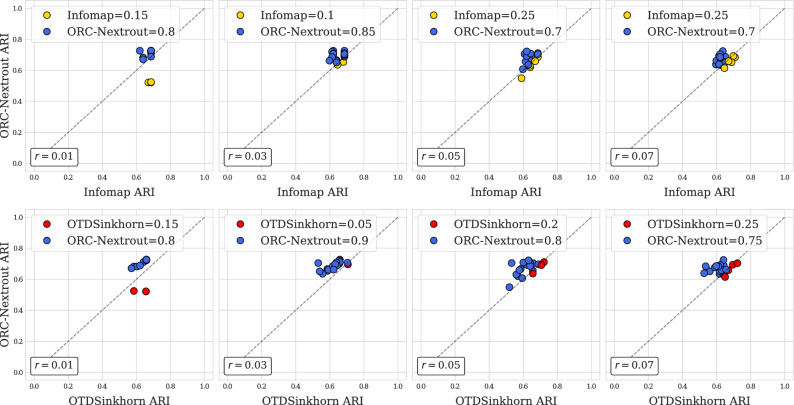
Removing intra-community edges test on Les Miserables data. Markers correspond to 20 instances of semi-synthetic networks generated from real data. Their (*x*, *y*) coordinates are ARI scores of the indicated method on the axes. Colors are given by the best performing algorithm, e.g. if $x>y$, the color of the associated method to *x* is chosen. The legend shows the percentage of times that the corresponding method outperforms the other. The parameter *r* describes the proportion of entries for the adjacency matrix *A* that have been changed. This increases from left to right.

We show the results obtained in the test of removing intra-community edges as scatter plots in Fig. 7. We use these plots to compare the algorithms on trial-by-trial community detection tasks: a point on each plot is an instance of a semi-synthetic network with (*x*, *y*) coordinates being the ARI scores of ORC-Nextrout (*y*) and either OTDSinkhorn or Infomap (*x*). If $y>x$, then ORC-Nextrout outperforms the other method in this particular dataset (blue markers), and vice versa if $x<y$. We then compute the percentage of times that ORC-Nextrout outperforms the other (as indicated in the legend). We find that ORC-Nextrout outperforms both OTDSinkhorn and Infomap clearly and consistently across different values of *r* ranging from $r=0.01 \ (R \approx 3)$ to $r=0.07 \ (R \approx 20)$. In at least 70% of the cases, ORC-Nextrout gives more accurate results than the other two algorithms. This suggests that ORC-Nextrout is more robust against perturbations of the community structure. Similar patterns are seen in the case where edges are removed at random and in semi-synthetic networks generated from the Dolphins dataset, see Supplementary Material.

## Conclusion

Community detection on networks is a relevant and challenging open area of research. Several methods have been proposed to tackle this issue, with no “best algorithm” that fits well for every type of data. We focused here on a recent line of work that exploits principles from Optimal Transport theory combined with the geometric concept of Ollivier-Ricci curvature applied to discrete graphs. Our method is flexible in that it tunes between different transportation regimes to extract the information necessary to compute the OR curvature on edges. On synthetic data, our model is able to identify communities more robustly than other OT-based methods based on the standard Wasserstein distance in the regime where inference is not trivial. On real data, our model shows better or comparable performance in recovering community structure aligned with node metadata compared to other approaches, thanks to the ability to tune the parameter $\beta$.

A relevant advantage of OT-based methods is that the number of communities is automatically learned from data, contrarily to other approaches that need this as an input parameter. In this respect, our model has the tendency to overestimate this number, similarly to other OT-based methods. Understanding how to properly incorporate small-size communities into larger ones in a principled and automatic way is an interesting topic for future work. Similarly, it would be interesting to quantify the extent to which various $\beta$ capture different network topologies. To address this, one could, for instance, use methods to calculate the structural distance between networks^74^ and correlate this against the values of the best performing $\beta$. Similarly, as our approach allows obtaining different sets of weights on edges, depending on $\beta$, it would be interesting to investigate how different values of this parameter impact network properties that are governed by the weight distribution, such as multi-fractality^75^.

There are a number of directions in which this model could be extended. Nodes can be connected in more than one way, as in multilayer networks. Our model could be extended by considering a different $\beta$ for each edge type, as done in^59,76^. Similarly, real networks are often rich in additional information, e.g. attributes on nodes. It would be interesting to incorporate a priori additional information to inform community detection^43,77^. This information can potentially be used to mitigate the problem of overestimation of the number of communities, as explained above. Finally, we have focused here on the flexibility of solving various transportation problems to provide different computations of the OR curvature. Different results could also be obtained by choosing different input mass distributions on nodes’ neighborhoods, as done in^33^. It would be interesting to combine these two approaches to reveal further insights of the role that the OR curvature plays in detecting communities in networks.

## Methods

### Optimal transport formulation

Consider the probability distributions *q* that take pairs of vertices and also satisfy the constraints $\sum _i q_{ij} = m_j, \sum _j \, q_{ij} = m_i$. In other words, these are the joint distributions whose marginals are $m_i$ and $m_j$. We call these distributions *transport plans* between $m_i$ and $m_j$. The Optimal Transport problem we are interested in is that of finding a transport plan $q^*$ that minimizes the quantity $\sum _{i\sim j} q_{ij}d_{ij} ,$ where $i\sim j$ means that the nodes *i* and *j* are neighbors and $d_{ij}$ is the cost of transporting mass from *i* to *j*, e.g. the distance between these two nodes. The quantity $\mathscr{W}_\beta (m_i,m_j,d):= \sum _{i,j} q^*_{ij} \,d_{ij}$, defined for this optimal $q^*$, is the *Wasserstein distance* between $m_i$ and $m_j$.

### The dynamical Monge–Kantorovich model

It was recently proved^51,52^ that solutions of the optimal transport problem previously stated can be found by turning that problem into a system of differential equations. This section is dedicated to describe this dynamical formulation.

Let $G=(V,E,W)$ be a weighted graph, with *N* the number of nodes and *E* the number of edges in *G*. Let $\mathbf{B}$ be the *signed incidence* matrix of *G*. Let $f^+$ and $f^-$ be two *N*-dimensional discrete distributions such that $\sum _{i\in V}f_{i}=0$ for $f = f^+-f^-$; let $\mu (t)\in \mathbb{R}^{E}$ and $u(t)\in \mathbb{R}^{N}$ be two time-dependent functions defined on edges and nodes, respectively. The discrete *Dynamical Monge-Kantorovich model* can be written as:

3 $$\begin{aligned} f_{i}&= \sum _{e} B_{ie} \frac{\mu _{e}(t)}{w_e} \sum _{j}B_{ej} \, u_{j}(t) , \end{aligned}$$

4 $$\begin{aligned} \mu _{e}'(t)&= \left[ \frac{\mu _{e}(t)}{w_e}\left| \sum _{j}B_{ej}\,u_{j}(t)\right| \right] ^{\beta }-\mu _{e}(t),\end{aligned}$$

5 $$\begin{aligned} \mu _{e} (0)&> {} 0, \end{aligned}$$

where $|\cdot |$ is the absolute value element-wise. Equation (3) corresponds to Kirchhoff’s law, Eq. (4) is the discrete dynamics with $\beta$ a traffic rate controlling the different routing optimization mechanisms; Eq. (5) is the initial distribution for the edge conductivities.

For $\beta = 1$ the dynamical system described by Eqs. (3)–(5) is known to reach a steady state, i.e., the updates of $\mu _e$ and $u_e$ converge to stationary functions $\mu ^*$ and $u^*$ as *t* increases. The flux function *q* defined as $q^*_{e}: = \mu ^*_{e} |u^*_{i}-u^*_{j}|/w_e$ is the solution of the optimal transport problem presented in the previous section. Notice that $\mu$ and *u* depend on the chosen traffic rate $\beta$, and thus, so does $q=q(\beta )$. Therefore, we can introduce a generalized version of the distance $\mathscr{W}$:

$$\begin{aligned} \mathscr{W}_\beta (m_i,m_j,w) := \sum _{i,j} q^*_{ij}(\beta )\,\,w_{ij}. \end{aligned}$$

We then redefine the proposed Ollivier-Ricci curvature as:

$$\begin{aligned} \kappa _\beta (i,j) := 1 - \dfrac{\mathscr{W}_\beta (m_i,m_j,w)}{d_{ij}}. \end{aligned}$$

### Probability distributions on neighborhoods

ORC-Nextrout takes in input a graph and a forcing term. While the graph encapsulates the neighborhood information provided by the nodes *i* and *j*, the forcing function is related to the distributions that one needs to transport. Analogously to what was proposed by^22^, we define this graph to be the weighted *complete bipartite* $B_{ij} = (V_{ij}, E_{ij}, \omega _{ij})$. The weights in $\omega _{ij}$ change iteratively based on the curvature. Notice that a bipartite graph must satisfy $\mathscr{N}(i)\cap \mathscr{N}(j) = \varnothing ,$ which does not hold true if *i* and *j* have common neighbors (this is always the case since $i\in \mathscr{N}(j)$). Nonetheless, this condition does not have great repercussions in the solution of the optimal transport problem since the weights corresponding to these edges (of the form (*i*, *i*)) are equal to 0. As for the forcing function, we define it to be $f := f^+ - f^- = m_i - m_j$.

### Other methods

To evaluate the performance of ORC-Nextrout, we compare with some of the well-established community detection algorithms including: Infomap^17^, MULTITENSOR^13^ (MT), discrete Ricci flow^22^ (OTDSinkhorn), Label propagation^14^ and Louvain^50^. We briefly describe each of these algorithms as follows;

- The *Discrete Ricci flow* (here addressed as OTDSinkhorn)^22^ is an iterative node clustering algorithm that deforms edge weights as time progresses, by shrinking sparsely traveled links and stretching heavily traveled edges. These edge weights are iteratively updated based on neighborhood transportation Wasserstein costs, similarly to what is proposed in this manuscript. After a predefined number of iterations, heavily traveled links are removed from the graph. Communities are then obtained as the connected components of this modified network.
- MULTITENSOR (MT)^13^ is an algorithm to find communities in multilayer networks. It is a probabilistic model with latent variables regulating community structure and runs with a complexity of *O*(*EK*) with assortative structure (as we consider here), where *K* is the number of communities. This model assumes that the nodes inside the communities can belong to multiple groups (mixed-membership). In this implementation we use their validity for single layer networks (a particular case of a multilayer network).
- *Infomap*^17^ employs information theoretic approach for community detection. This method uses the map equation to attend patterns of flow on a network. This flow is simulated using random walkers’ traversed paths. Based on the theoretic description of these paths, nodes with quick information flow are then clustered into the same groups. The algorithm runs in *O*(*E*). In the experiments, we fix the number of random initialization of the random walkers to be equal to 10. The inferred partition is then the one minimizing the entropy.
- *Label propagation*^14^ assigns each node to the same community as the majority of its neighbors. Its working principle start by initializing each node with a distinct label and converges when every node has same label as the majority of its neighboring node. The algorithm has a complexity scaling as *O*(*E*).
- *Louvain*^50^ is a fast algorithm used to find communities on networks by maximizing the modularity of the associated partitions. It consists of two phases. First, it assigns every node on the network into a different community. Then, it aggregates nodes and neighbors based on gains of modularity. This last step is repeated until no further improvement can be achieved.

## Supplementary Information

Supplementary Information.

## Data Availability

The real datasets analyzed during the current study are available at http://www-personal.umich.edu/~mejn/netdata/. The synthetic data generated are available from the corresponding author upon request.

## Code availability

Open source codes and executables are available at https://github.com/Danielaleite/ORC-Nextrout. https://gitlab.com/enrico_facca/dmk_solver.
