# Supplementary material for: Community detection in networks by dynamical optimal transport formulation
Source: Sci Rep. 2022 Oct 7;12:16811. doi: 10.1038/s41598-022-20986-y (PMC9546897; doi:10.1038/s41598-022-20986-y)
Supplement: Supplementary file 1 — Supplementary Information. [file 41598_2022_20986_MOESM1_ESM.pdf]

Supplementary information for
Community Detection in networks by Dynamical Optimal Transport Formulation

Daniela Leite[1,*,+], Diego Baptista[1,*], Abdullahi A. Ibrahim[1], Enrico Facca[2], and Caterina De Bacco[1]

[1]Max Planck Institute for Intelligent Systems, Cyber Valley, 72076 Tübingen, Germany
[2]Univ. Lille, Inria, CNRS, UMR 8524 - Laboratoire Paul Painlevé, F-59000 Lille, France
[+]daniela.leite@tuebingen.mpg.de
[*]These authors contributed equally to this work

Supplementary Note 1: Real networks

This section presents additional plots for real networks on communities detected by some algorithms studied in this paper. Supplementary Fig. 1a and Supplementary Fig. 1b exhibit the detected communities for American football and Political books, respectively. For each dataset we compare three algorithms with the ground truth, and with ORC-Nextrout having a single setting of β.

(a) American Football

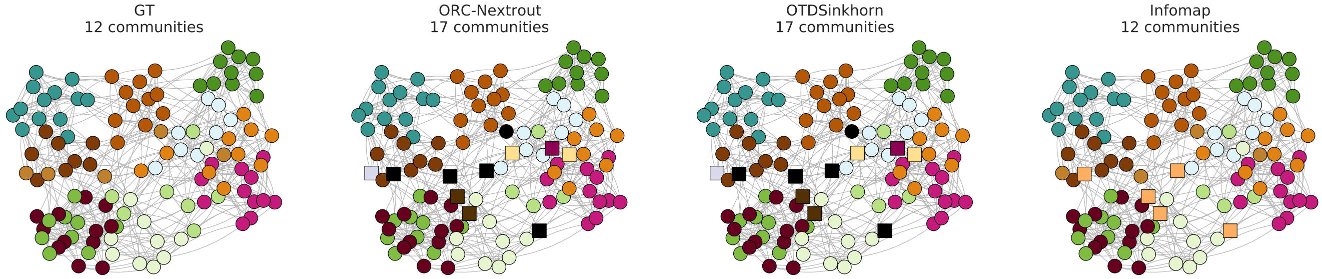

(b) Dolphins

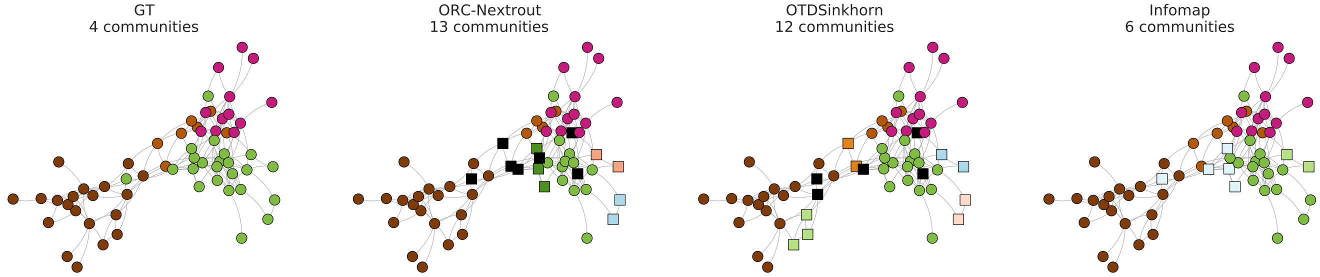

Supplementary Figure 1. Communities in real networks. We show the communities for American football (a) and Dolphins (b) inferred by ORC-Nextrout ($\beta = 2.0, 1.5$ for top and bottom rows respectively), OTDSinkhorn and Infomap and compare against those extracted using node attributes (GT). Dark nodes represent individual nodes who are assigned to isolated communities by OT-based methods. Square-shaped markers denote nodes assigned to communities different than those obtained from node metadata.

Supplementary Note 2: ARI score

We dedicate this section to present the main score used in the experiments described in this manuscript.
The *Rand Index*[1] (RI) is a measure of the similarity between two sets of clusters. Let $S = \{x_1, x_2, ..., x_n\}$ be a set of n different elements, and let $X = \{X_1, X_2, .., X_m\}$, $\hat{X} = \{\hat{X}_1, \hat{X}_2, .., \hat{X}_p\}$, be two partitions of S into m and p subsets, respectively. Intuitively,

X can thought of as the "correct" separation of S into classes, while $\hat{X}$ would be a "prediction" of it. We would like to understand the quality of the prediction $\hat{X}$ in terms of the ground truth community information X. Consider

- TP (*true positive*) is the number of times that a pair of elements (x_i, x_j) belonging to the **same** class X_k gets assigned to the **same** class $\hat{X}_l$.
- TN (*true negative*) is the number of times that a pair of elements (x_i, x_j) belonging to **different** classses in X gets assigned to **different** classes in $\hat{X}$.
- FP (*false positive*) is the number of times that a pair of elements (x_i, x_j) belonging to **same** class X_k is (*falsely*) assigned to **different** classes $\hat{X}_l$ and $\hat{X}_m$.
- FN (*false negative*) is the number of times that a pair of elements (x_i, x_j) belonging to **different** classes X_k and X_l is (*falsely*) assigned to the **same** class $\hat{X}_m$.

One can think of the the words *positive* and *negative* referring to whether two elements in S belong to the same or to different classes, respectively. The words *true* and *false* would then judge the performance of the prediction: if it maches the nature of the elements under inspection, then the word *true* is associated to it; *negative*, otherwise.

The Rand Index is then computed using the formula:

$$RI(X, \hat{X}) = \frac{TP + TN}{TP + FP + FN + TN}.$$

The *Adjusted Rand Index* (ARI) is the *corrected for chance* version of the RI:

$$ARI(X, \hat{X}) = \frac{RI - \mathbb{E}[RI]}{1 - \mathbb{E}[RI]},$$

where $\mathbb{E}[RI]$ is the expected value of the RI under the assumptions that the partitions X and $\hat{X}$ are sampled from the generalized hypergeometric distribution. Closed forms for the terms shown in the ARI formula can be computed. See[1] for a more detailed presentation of the RI and ARI scores.

Supplementary Note 3: Two tests on random networks generated from real structures

Algorithm and adjacency matrix

The pseudo-code of the random processes defined on Section ***Two tests on semi-synthetic networks*** of the manuscript is shown in the Algorithms 1 and 2.

Algorithm 1 Flipping entries of the adjacency matrix

```
Input: G = (V, E, W), flipping proportion r ∈ [0,1], flipping probability p ∈ [0,1]
Output: G' = (V, E')
Build adjacency matrix of G: A
Make a copy of A: A'
Compute number of nodes in G: N
for t ∈ range(r ∗ N^2) do
  for i ∈ range(N) do
    for j ∈ range(i+1, N) do
      Assume P_ij(A[i][j]) = p
      Sample A'[i][j] from P
    end for
  end for
end for
Symmetrize A'
Build G' using the connections between nodes described by A'.
```

We show two examples of the outputs of these algorithms on Supplementary Figure 2 together with the adjacency matrix that was used to originally build them. Notice that the matrix in panel (b) is different from that in panel (a) both in terms of intra and inter-community blocks, whereas that in (c) only differs along the within-community entries. This indicates that the first method alters the overall configuration of the edges in the network by adding random noise, whereas the second changes the original network only by reducing the intra-community relationships.

Algorithm 2 Removing intra-community edges

Input: $G = (V, E, W)$, removal proportion $r \in [0, 1]$,
Output: $G' = (V, E')$
Build list of edges of G: $E(G)$
Make a copy of E: $E'(G)$
Compute number of edges in G: M
Remove from $E'(G)$ all the edges $e = (i, j)$ such that either i or j is a leaf in G
Remove $r * M$ elements from $E'(G)$ uniformly at random
Define G' using V and $E'(G)$

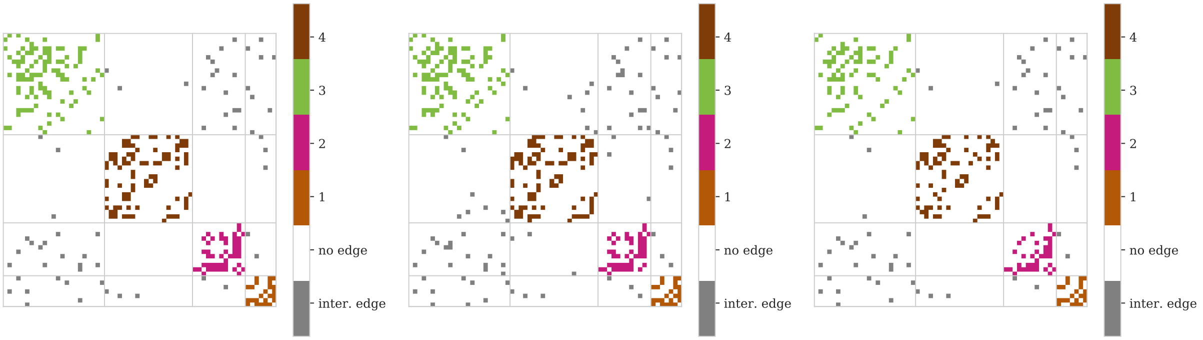

(a) Original adjacency matrix **(b)** Flipping entries **(c)** Removing intra-community edges

Supplementary Figure 2. Adjacency matrices for original and perturbed Dolphins network. We show the adjacency matrix of (a) the original dataset, (ii) a perturbed network built from the previous one by flipping entries at random and (c) a perturbed graph obtained by removing intra-community edges. We used $r = 0.05$ and $r = 0.15$ to generate the matrices shown in (b) and (c), respectively. Nodes are grouped by communities to highlight the block structure. Gray lines denote the boundaries of these blocks. Diagonal blocks represent the communities. Off-diagonal blocks show connections between communities. Colored entries are in agreement with those of community layout shown in Supplementary Fig. 1b. Inter-community connections are highlighted in gray.

Results

We show results obtained on the Les-Miserables dataset for the test where we flip at random the entries of the adjacency matrix A (see Supplementary Figure 4). In this case, ORC-Nextrout outperforms Infomap only in the case where $r = 0.005$, i.e. when 5% of elements of A are changed. As r increases, Infomap increases its accuracy. On the other hand, ORC-Nextrout shows a better performance than OTDSinkhorn consistently across values of r.

Lastly, in Supplementary Figure 4. we show the results obtained for both tests on the Dolphins dataset. It can be seen that ORC-Nextrout outperforms OTDSinkhorn consistently across values of r in both cases. On the other hand, Infomap has a higher accuracy in both tests, as expected given the results shown in Figure 5 of the main manuscript.

References

1. Hubert, L. & Arabie, P. Comparing partitions. *J. classification* **2**, 193–218 (1985).

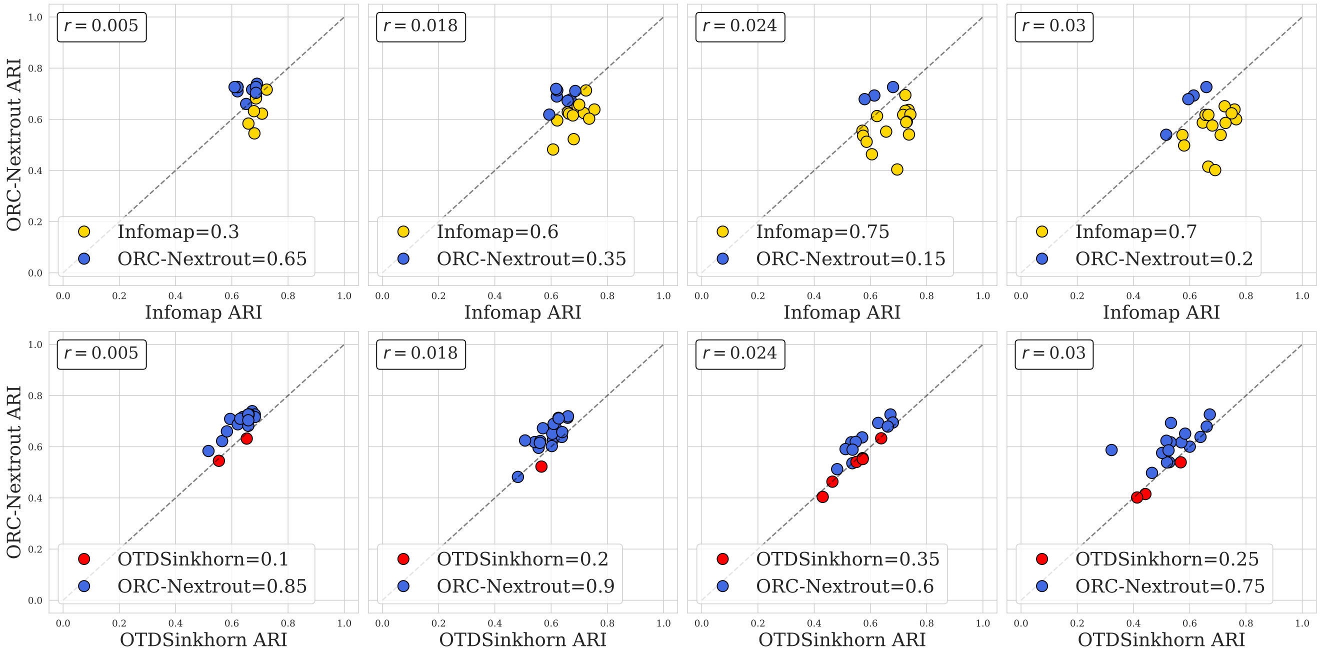

Supplementary Figure 3. Flipping-entries test on Les Miserables data. Markers correspond to 20 instances of semi-synthetic networks generated from real data. Their (x, y) coordinates are the ARI scores of the method indicated on axes. Colors are given by the best performing algorithm, e.g. if $x > y$, the color of the method associated to x is chosen. The legend shows the percentage of times that the corresponding method outperforms the other. The parameter r describes the proportion of entries of the adjacency matrix A that have been changed. This increases from left to right.

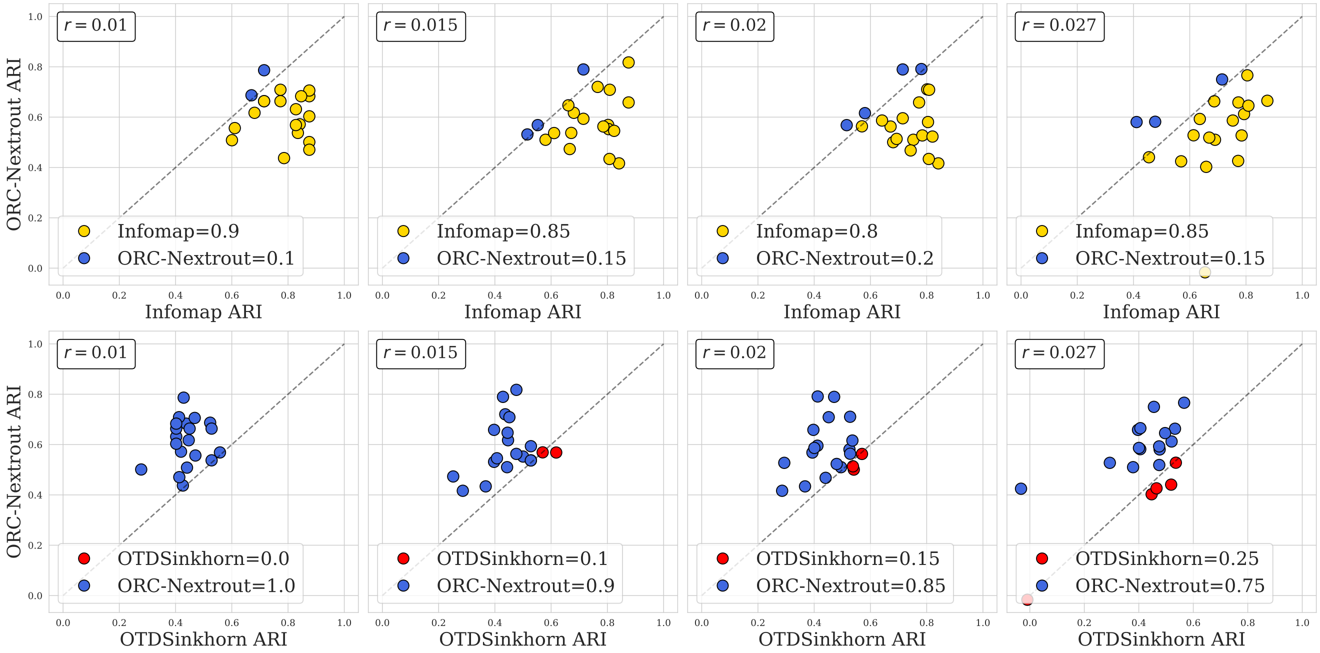

Supplementary Figure 4. Flipping-entries test on Dolphins data. Markers' description is similar as in Supplementary Figure 3.

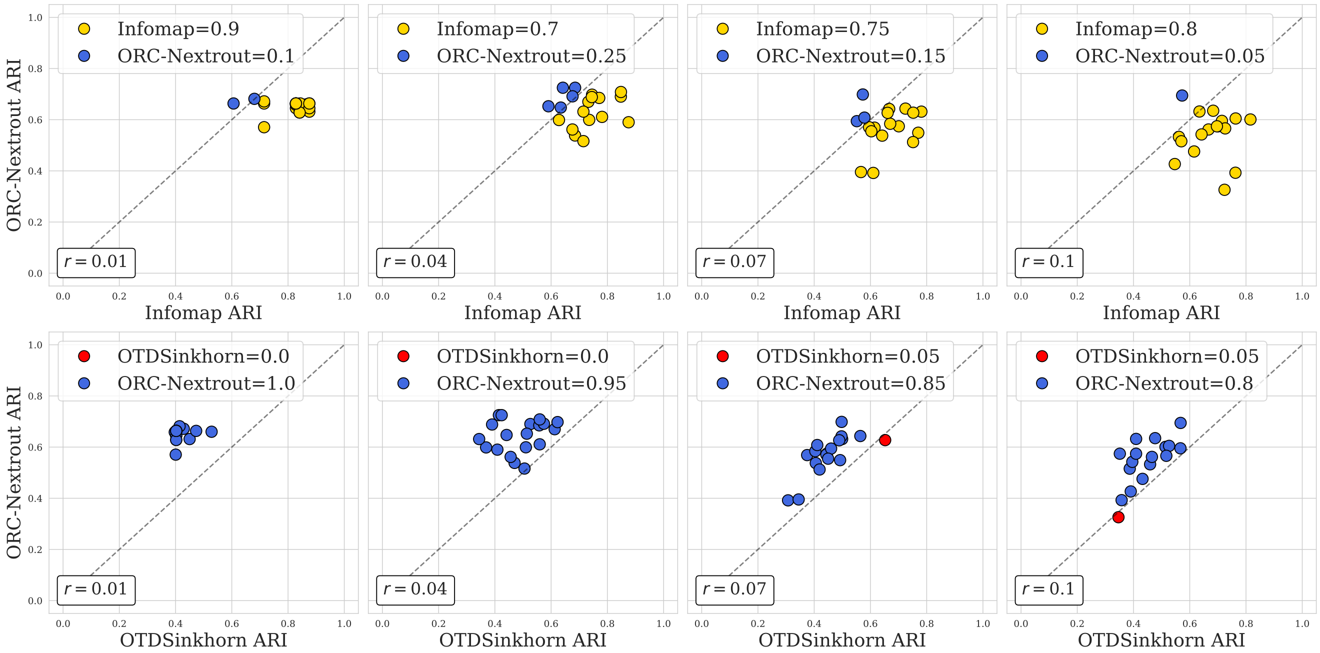

Supplementary Figure 5. Removing intra-community edges test on Dolphins data. Markers' description is similar as in Supplementary Figure 3.
